# Exploring cognitive function and postoperative neurocognitive recovery after cardiac surgery in older adults (ECPON): a protocol for an observational study

**DOI:** 10.1136/bmjopen-2024-098208

**Published:** 2025-06-22

**Authors:** Lina Bergman, Ernad Zecevic, Tor Damén, Gabriela Markovic, Anna Martinik, Markus Saarijärvi, Jeanette Eckerblad, Ulrica Nilsson

**Affiliations:** 1 Department of Neurobiology, Care Sciences and Society, Karolinska Institutet, Huddinge, Sweden; 2 Perioperative medicine and intensive care, Karolinska University Hospital, Huddinge, Sweden; 3 Department of Anaesthesiology and Intensive Care Medicine, Institute of Clinical Sciences, Sahlgrenska Academy, University of Gothenburg, Gothenburg, Sweden; 4 Department of Cardiothoracic Anaesthesia and Intensive Care, Sahlgrenska University Hospital, Gothenburg, Sweden; 5 Department of Rehabilitation Medicine, Danderyd University Hospital, Stockholm, Sweden; 6 Department of Clinical Sciences, Karolinska Institutet, Stockholm, Sweden; 7 Department of Cardiology, Danderyd University Hospital, Stockholm, Sweden

**Keywords:** Thoracic surgery, Cardiovascular Disease, Delirium & cognitive disorders

## Abstract

**Abstract:**

**Introduction:**

Cardiovascular disease is one of the most common health issues facing the older population, and the number of older adults undergoing cardiac surgery is expected to increase. Postoperative neurocognitive impairment is a frequent and often unrecognised complication that can adversely affect a patient’s recovery, quality of life and daily activities, as well as impact the lives of their family members. Patients may express cognitive difficulties as a feeling of ‘not being the same since the operation’. This study aims to investigate the factors that influence neurocognitive function and patient-reported cognitive symptoms among patients aged 65 and older following cardiac surgery, and explore the impact on the overall postoperative recovery. Additionally, the study aims to describe the perspectives of close relatives on the recovery process.

**Methods and analysis:**

A longitudinal observational study with a mixed-methods approach will be conducted in two thoracic surgical departments in Sweden. A total of 220 patients and 1 close relative for each patient will participate. Neurocognitive function will be assessed preoperatively and at 1, 3 and 6 months postoperatively using a digitalised neurocognitive test battery. We will also evaluate postoperative patient-reported cognitive symptoms and signs, delirium, frailty, health-related quality of life, depression, perceived self-efficacy, fatigue and functional capacity. Each patient’s close relative will assess the observed cognitive function and report on caregiver burden. At the 6-month mark, a purposive sample of patients and their close relatives will be interviewed to explore their experiences of postoperative cognitive recovery.

**Ethics and dissemination:**

The study has been approved by the Swedish Ethical Review Authority (Reference number: 2024-03380-01) and will adhere to the Helsinki Declaration and its amendments. The results will be disseminated through peer-reviewed journals and scientific conferences, as well as presented in various popular science forums and patient organisations.

**Trial registration number:**

NCT06469515; Pre-results.

**STRENGTHS AND LIMITATIONS OF THIS STUDY**

- This study employs a novel approach to assess neurocognitive function through a digital self-administered neurocognitive test battery.
- By employing a mixed-methods approach, the study provides a comprehensive, reliable and flexible analysis, contributing to a more complete and nuanced understanding of cognitive recovery following surgery, particularly from the patient’s own perspective.
- Incorporating the experiences and perspectives of close relatives will enhance our understanding of neurocognitive function and the process of returning to everyday life after cardiac surgery.
- However, the study has a high response burden, which increases the risk of participant dropouts and may impact the quality of the data collected.

## Introduction

 The global population of older adults is increasing rapidly, making this demographic group the fastest-growing worldwide. By 2070, it is projected that one in four people in Sweden will be 65 years or older,^1^ and similar trends can be observed globally.^2^ As cardiovascular disease is one of the most common health issues among older adults, it is anticipated that the number of older adults undergoing cardiac surgery will also rise.^3 4^ Older adults are more susceptible to postoperative complications, which are often medical rather than surgical in nature. They tend to experience longer hospital stays and higher rates of readmission compared with the general population.^5^ Additionally, many older adults face multimorbidity,^6^ and a significant number may be classified as frail,^7^ whether or not they have pre-existing cognitive impairments.^8^

Postoperative neurocognitive impairment is a frequent yet often unrecognised complication following cardiac surgery, which can affect the patient’s recovery, quality of life and daily functioning, as well as impact their close relatives.^9^ Perioperative neurocognitive disorder, previously known as postoperative cognitive dysfunction, is an overarching term used to describe cognitive impairment that occurs post-surgery, reflecting a decline in neurocognitive performance from the preoperative baseline.^10^ This includes postoperative delirium (POD), characterised by a sudden decline in attention and cognitive function occurring within 7 days after surgery, delayed neurocognitive recovery (dNCR), which refers to cognitive dysfunction that persists or arises within 30 days after the procedure, as well as mild or major postoperative neurocognitive disorders (p-NCD) that can be identified up to 12 months after surgery.^10^ To diagnose dNCR and p-NCD, the Diagnostic and Statistical Manual of Mental Disorders, Fifth Edition pillars of diagnosis for neurocognitive disorders^11^ are required: (1) subjective complaint/cognitive symptoms assessed by the patient, a caregiver or a close relative/friend; (2) objective impairment/change assessed by neurocognitive tests and (3) the individual’s functional level related to the activities of daily living.^10^

Previous research demonstrates that approximately 40%–50% of patients undergoing cardiac surgery develop cognitive complications in the early postoperative period.^9^ The aetiology of these complications is believed to be multifactorial, with the proposed mechanisms, including embolisation, neural ischaemia and inflammatory response.^12^ It has been suggested that the use of cardiopulmonary bypass during cardiac surgery also affects cognitive outcomes; however, no definitive conclusions have been reached.^13^ Several risk factors for developing dNCR/p-NCD have been identified, including advanced age, pre-existing cognitive impairment, preoperative depression and frailty, low educational attainment, atherosclerotic cardiovascular diseases, diabetes, prolonged mechanical ventilation and extended surgery duration.^14^,^18^ Currently, routine clinical evaluations of cognitive function are not a part of the preoperative or postoperative assessments. Therefore, knowledge about the range of patient-reported cognitive symptoms and signs that patients may experience, the timing of these events, and their impact on daily life remains limited.

When cognitive function declines, it significantly impacts the patient’s close relatives, resulting in a decreased quality of life and increased caregiver burden.^19^ Close relatives are often the first to notice changes in a patient’s cognitive status.^20^ In this study, the term ‘close relatives’ refers to all individuals identified by the patient as significant to them, which may include close friends and other important figures in their life. These relatives may play a crucial role in diagnosis and support the patient’s recovery process. These aspects have not yet been extensively studied. If patient-reported cognitive symptoms are effectively assessed and managed, it may lead to the improved quality of life for both the patient and their close relatives.

### Aim

The purpose of this study is to investigate the factors that influence neurocognitive function and patient-reported cognitive symptoms among patients aged 65 and older following cardiac surgery, and explore the impact on the overall postoperative recovery. Additionally, the study aims to describe the perspectives of close relatives on the recovery process. The following scientific questions will be addressed in the study.

1. What is the incidence of dNCR/p-NCD and the occurrence of patient-reported cognitive symptoms in older adults up to 6 months after cardiac surgery?
2. Is there a correlation between preoperative cognitive status and dNCR/p-NCD, and if so, which cognitive function (executive function, attention and memory) is most affected?
3. Is there a correlation between POD and dNCR/p-NCD, and if so, which cognitive function is most affected?
4. Which factors (eg, age, educational level, duration of surgery, perceived self-efficacy, frailty and mental health and depression) affect the risk of suffering from postoperative cognitive decline?
5. Is dNCR/p-NCD associated with increased symptom burden, impaired functional level, fatigue, frailty, deteriorated quality of life and an increased number of unplanned contacts with healthcare services?
6. How do older adults with persistent cognitive symptom burden experience their postoperative recovery and return to daily life?
7. How do close relatives to older adults with persistent cognitive symptom burden perceive the patient’s postoperative cognitive function and recovery, and how does this affect the relative’s own life?

## Methods and analysis

### Study design and setting

A longitudinal observational study using a mixed-method convergent parallel design will be conducted at two thoracic surgical departments in Sweden.^21^ The study will adhere to the STrengthening the Reporting of OBservational studies in Epidemiology guidelines.^22^ Recruitment for the study began in November 2024 at the first site and in March 2025 at the second site.

### Participants

Participants in the study will consist of older adults aged 65 years and above who are undergoing open cardiac surgery (eg, bypass grafting or valve replacement or a combination of both). The goal is to include a total of 220 patients, along with one accompanying close relative for each patient. The sample size estimation is based on a statistical power analysis aimed at detecting a 25% rate of dNCR among participants 1 month after surgery, using a normal SD with α=Zα = 1.960 and factoring in a 15% dropout rate.

#### Inclusion criteria

- Scheduled for elective or urgent cardiac surgery.
- Resident in the area/region for the current thoracic clinic.
- Able to read and understand Swedish.

#### Exclusion criteria

- Participants with physical, mental or cognitive difficulties preventing them from completing the cognitive tests.
- Individuals who have undergone surgery within the last 6 months.

The close relative will be eligible for inclusion if (1) the relative is 18 years or older and (2) can read and understand Swedish.

### Recruitment

Potential participants (patients) will be assessed for eligibility and provided with both written and verbal information about the study. Following this, they will be asked to participate. If a patient chooses to be included, a close relative will also be invited to participate in the study. If the patient does not have any relatives or their close relatives choose not to participate, they will not be excluded from the study. Close relatives will receive written and verbal information about the study and will, subsequently, be enrolled after providing informed written consent. Recruitment will be conducted by a research nurse at each study site.

### Outcome measure

#### Patient-reported cognitive symptoms and signs

The Swedish Quality of Recovery cognitive version (SwQoR-Cog) is a self-assessment questionnaire designed to measure postoperative symptoms. The SwQoR-Cog has been developed from the original SwQoR, which measures general postoperative recovery.^23^ In the cognitive version, the number of items has been reduced, and additional items focusing on patient-reported cognitive symptoms and signs have been included. This refinement in targeting cognitive recovery is based on the previous research,^24^,^26^ clinical experience and our ongoing research in the field of neurocognitive recovery.^27^

The SwQoR-Cog consists of 14 statements regarding symptoms and discomfort. Of these, 10 address cognitive symptoms (eg, fatigue, difficulties in sleeping, reading/calculating and concentrating), while 4 pertain to general postoperative recovery. The symptoms are rated on an 11-point numerical scale, ranging from 0 (‘not at all’) to 10 (‘all the time’).

#### Neurocognitive function

A digital neurocognitive test battery (Mindmore-P) will be used to assess neurocognitive function, specifically measuring verbal episodic memory and executive functions, including visuospatial skills and selective attention (table 1). This digital test battery is self-instructive and accessible through an online platform. It is designed to be comparable to the traditional analogue International Study Group of Postoperative Cognitive Dysfunction test battery, and participants have reported a high level of usability.^28^ Preliminary results indicate that the digital test battery is feasible for use in clinical settings with older adults who have undergone major abdominal surgery (manuscript submitted). Before undergoing neurocognitive tests, the patient will evaluate their vision and hearing and rate their level of restfulness and stress using a five-point scale.

**Table 1:** Included test in Mindmore-P

Test	Cognitive domain and function	Description
CERAD	Verbal episodic memory	Used to assess verbal learning skills, immediate recognition and delayed recognition. The participant is presented with ten words and then asked to repeat the words at different timepoints. At one point, the participant will be presented with a larger list of words and asked to select the correct words.
TMT A and B	Executive, visuospatial and processing speed	Measures mental and motor speed, and mental flexibility. This test requires the use of an external computer mouse for the most accurate result. The participant will be presented with letters and digits and asked to connect them in a specific order as fast as possible.
SCW	Executive, selective attention, cognitive flexibility and inhibitory control	Assesses for inhibitory control by presenting words written in different colours and asking for either naming the correct colour or naming the colour of a printed incongruent colour word. The test is time-sensitive and requires both speed and accuracy.
SDPT	Executive, visuospatial, processing speed, concentration and verbal learning	The test relies on psychomotor speed and the ability to decipher symbols. The participant is presented with symbols that represent digits as a code and then asked to click on the correct digit as many times as possible.

CERAD, consortium to establish a registry for Alzheimer’s disease; SCW, stroop colour-word test; SDPT, symbol digits processing test; TMT, trail making test (A and B).

#### Delirium

Delirium screening will be conducted using the nursing delirium scale (Nu-DESC).^29^ This scale evaluates five areas to assess the presence and severity of symptoms in patients. It uses a 3-point grading scale, where a total score of 2 or higher indicates the presence of delirium. The Nu-DESC has been validated for use with older adults in a Swedish context.^30^

#### Depression

The patient health questionnaire-9 (PHQ-9) will be used to assess depressive symptoms.^31^ This questionnaire consists of nine statements to which respondents should respond on a four-point scale. A score of 0 indicates ‘not at all’, while a score of 3 means ‘almost every day’. Respondents should consider their experiences over the past 2 weeks when answering. The PHQ-9 is a validated tool for detecting and monitoring depression.^32^

#### Frailty

The clinical frailty scale (CFS)^33^ is a validated and reliable assessment tool used for evaluating a person’s frailty. It consists of nine levels ranging from (1) very vital to (9) terminally ill. This objective assessment is conducted by trained professionals based on the individual’s habitual status prior to illness. The CFS has been validated in a Swedish context.^34^

#### Self-efficacy

The general perceived self-efficacy scale^35^ is a ten-item questionnaire that allows respondents to answer on a four-point scale. It measures the respondents’ belief in their ability to face challenges and resolve problems. The instrument has undergone validity testing in Sweden.^36^

#### Health-related quality of life

The RAND-36 will be used to assess health-related quality of life. This instrument consists of 36 questions divided into 8 domains: physical function, limitations due to physical health, limitations due to emotional problems, energy and fatigue, emotional well-being, social functioning, pain and general health. In addition to the total score, two summary scores will be calculated: the physical component summary and the mental component summary. These summaries reflect the overall physical and mental health status. The RAND-36 has been translated into Swedish and has been reliability tested.^37^

#### Functional capacity

The WHO disability assessment schedule 2.0^38^ measures an individual’s level of daily activity. It consists of 12 questions regarding the difficulty of completing everyday activities in relation to the respondent’s current health. Respondents can answer on a 5-point scale, with scores ranging from 0 to 4. Higher scores indicate increased disability.^39^

#### Fatigue

The fatigue scale for motor and cognitive functions measures fatigue in relation to cognitive and physical functioning. This questionnaire consists of 20 statements regarding how fatigue impacts a person’s everyday life. Respondents can rate each statement on a five-point scale, with higher scores indicating more severe fatigue. The assessment results will categorise fatigue as mild, moderate or severe.^40^

#### Cognitive assessment of close relatives

The cognitive failures questionnaire for others (CFQ-f) is designed for close relatives to assess cognitive difficulties observed in their loved ones during daily activities.^41^ It consists of eight questions, each rated on a five-point scale ranging from ‘never’ to ‘always’. This questionnaire has been translated into Swedish by our research group using back-translation techniques. It has also been adapted for the Swedish context to ensure that the wording is appropriate for the Swedish language and culture. The timeline has been modified to focus on the past month rather than the last 6 months, as in the original questionnaire, to better align with the recovery process.

#### Caregiver burden

The short form of the Zarit Burden Interview (ZBI-12) will be used by close relatives to evaluate their perceived burden.^42^ It comprises 12 questions, with responses rated on a 5-point scale from ‘never’ to ‘almost always’. The ZBI-12 short form has been tested for validity and reliability in the Swedish context.^43^

#### Demographic and perioperative data

This will include sociodemographic and perioperative data, such as civil status, educational level, hearing and visual impairments, comorbidities, American Society of Anesthesiologists (ASA) classification, duration of surgery and anaesthesia, other clinical characteristics and postoperative complications. Any unplanned health visits will be documented at the endpoint.

#### Experiences of postoperative cognitive recovery

To gain a better understanding of the recovery process for older adults following cardiac surgery, specifically regarding cognitive recovery, qualitative interviews will be conducted with patients and their close relatives. The semi-structured interview guide for patients will include questions, such as: ‘can you describe what a typical day has been like for you since the surgery?’ and ‘have you experienced any patient-reported cognitive symptoms or difficulties since the surgery, and what do they entail?’ The interview guide for close relatives will feature questions like: ‘can you share your experiences since the surgery and how it has impacted you and (the name of the person who underwent the surgery)?’ and ‘how has the recovery process affected your daily life?’

### Procedure

#### Preoperative data collection

Before surgery, the patient’s baseline data will be collected approximately 14 days in advance, following their informed written consent to participate in the study. We will measure patient-reported cognitive symptoms, self-efficacy, frailty (if it has not been routinely recorded at the hospital site), fatigue, health-related quality of life and collect demographic data. This data collection can take place digitally, at the hospital or through home visits. The process of collecting data will be thoroughly documented.

All questionnaires will be distributed using the Research Electronic Data Capture (REDCap) system. If participants prefer, the data can also be collected using paper-and-pen questionnaires or through phone assessments. Neurocognitive function will be evaluated using the Mindmore-P tool on a digital platform. Each test begins with an introduction, followed by a practice trial and then the actual test trial, which takes approximately 15–20 min to complete. Although Mindmore-P is self-instructive, a trained test leader (such as a research nurse or researcher) will be available by phone or digital platform for distance assessments, or in person at the hospital, to provide support and answer any questions that may arise. Close relatives of the participants will be asked, after providing their consent, to provide demographic information and respond to questionnaires about the patient’s cognitive function and caregiver burden prior to surgery. Table 2 outlines the outcome measures and the time points for assessment.

**Table 2:** Outcome measurements and timepoint for assessment

Outcomes	Instrument	Timepoint of measurement					
		Preop	Postop day 1–7	At discharge	1 month	3 months	6 months
Participant (patient)							
Cognitive symptoms	SwQoR-Cog	X		Every seventh day → → → → → →→→→			X
Neurocognitive function	Mindmore-P	X			X	X	X
Delirium	Nu-DESC		X				
Frailty	CFS	X					X
Fatigue	FSMC	X					X
Self-efficacy	S-GSE	X			X	X	X
Depression	PHQ-9	X			X	X	X
Health-related quality of life	RAND-36	X					X
Functional level	WHODAS 2.0	X			X	X	X
Experience of postop recovery	Interview						X
Unplanned hospital visits	Study specific						X
Demographical data	Study specific	X					
Surgical and postop data	Patient record			X			
Participant (close relative)							
Cognitive assessment for others	CFQ-f	X			X	X	X
Assessment of perceived burden	ZBI-12	X			X	X	X
Experience of postop recovery	Interview						X

CFQ-f, cognitive failures questionnaire for others; CFS, clinical frailty scale; FSMC, fatigue scale for motor and cognitive functions; GSE, general perceived self-efficacy scale; Nu-DESC, nursing delirium scale; PHQ, patient health questionnaire; SwQoR-Cog, Swedish quality of recovery cognitive version; WHODAS, WHO disability assessment schedule; ZBI, Zarit burden interview.

#### Postoperative data collection and follow-up

After surgery, participating patients will be monitored for postoperative delirium from postoperative day 1 to postoperative day 7, or until they are discharged from the hospital. Following this period, they will be asked to evaluate their cognitive symptoms weekly for up to 3 months after surgery. The questionnaire will be distributed digitally using REDCap (table 2).

At both 1-month and 3-month post-surgery, we will assess neurocognitive function (using the Mindmore-P), self-efficacy, depression and functional capacity. At the 6-month mark (the endpoint of the study), we will also evaluate frailty, fatigue and health-related quality of life. Perioperative data will be collected from the patient’s medical records. Close relatives of the patients will complete the same questionnaires (focused on the cognitive assessment of the relative and caregiver burden) at 1-month, 3-month and 6-month postsurgery. Each follow-up session is expected to take approximately 40–50 min for the patients and about 10 min for the close relatives (table 2).

#### Qualitative interviews

Qualitative semistructured interviews will be conducted approximately 6 months after the surgery with a subsample of participants. Both patients (n≥20) and their close relatives (n≥20) will be invited to participate, and they will be interviewed separately to capture their individual experiences. The estimated number of participants for qualitative interviews is informed by the concept of information power (ie, the expected amount of information required to answer the research question).^44^ We aim to include patients who have experienced cognitive symptoms. To identify these individuals, we have established cut-off scores for persistent cognitive symptom burden. Cognitive symptom burden is assessed using the SwQoR-Cog, with four non-cognitive items removed. The remaining 10 items form the cognitive subscore, ranging from 0 to 100. The threshold for symptom burden is defined as a subscore of ≥30. For inclusion in qualitative interviews, participants must exceed the cut-off (ie, ≥30) measured at 6 and 12 weeks postoperatively. If the patient has undergone another major surgery between the cardiac surgery and the interview, they will not be considered for the interview. Major surgery is defined as a surgical procedure that lasts longer than 1 hour in duration. Participants will have the option to choose the interview location, which can be face-to-face, digital or via telephone. The interviews are expected to last between 30 and 60 min (table 2).

### Data analysis and statistics

Descriptive statistics will be presented, including frequency, percentage, mean, SD or range, as appropriate. We will calculate standardised reliable change Z-scores for each neurocognitive test (n=4) based on the differences in patients' test results from baseline to postoperative assessments, adjusting for variance using Swedish normative data.^45^ In accordance with the guidelines established by Borchers *et al*. in their 2021 review^46^ and Evered *et al*. in their 2018 nomenclature,^10^ we define objective decline in dNCR/p-NCD as mild (1–2 SD) and major (greater than 2 SD) for each test result. This classification requires at least two test results for accuracy.

Parametric and non-parametric tests will be used to identify differences between groups regarding outcomes and demographic characteristics. We will assess potential associations between our dependent variable (neurocognitive performance) and independent variables using multivariate logistic regression analysis. A p value of less than 0.05 will be considered statistically significant in all analyses.

Mixed effect models will be used for the analysis of longitudinal data and potential clustering of data on patient-reported cognitive symptoms. Trajectories of patient-reported cognitive symptoms will be analysed with statistical process control (SPC).^47^ The SPC involves the creation of control charts that are used to evaluate how processes change over time. A pooled SD measure based on the first two trials from SwQoR-Cog will be used for the calculation of control limits. X-bar and S charts will be used for process analysis on group level. Individual processes will be analysed with I-diagrams within SPC.

The SwQoR-Cog and CFQ-f will undergo further psychometric evaluation, guided by the COnsensus-based Standards for the selection of health Measurement INstruments.^48^ Additionally, interviews with patients and their close relatives will be analysed using qualitative content analysis, which is appropriate for exploring and identifying knowledge where evidence is limited.^49^ Finally, we will synthesise both quantitative and qualitative findings to identify similarities and differences, examining whether the qualitative results converge with or diverge from the quantitative findings.^21^

## Ethics and dissemination

The study has received approval from the Swedish Ethical Review Authority (reference number: 2024-03380-01) and will comply with the Helsinki Declaration and all its amendments. All participants will be thoroughly informed about the study, including details regarding confidentiality, voluntary participation and the requirement for written consent before enrolment. The results from the neurocognitive tests will not be shared with anyone, including the participants themselves. Additionally, information obtained from participants will not be disclosed to their close relatives, and vice-versa. We will follow good clinical practice in the conduct of clinical trials. The findings from the study will be published in peer-reviewed journals and presented at scientific conferences, as well as in various popular science forums.

### Patient and public involvement

Patients or the public were not involved in the design, or conduct, or reporting or plans of this research.

## Discussion

This study aims to investigate neurocognitive function and symptom burden in older adults following open cardiac surgery, as well as to describe the perspectives of their close relatives. Our society is ageing, and as life expectancy increases, the number of older individuals continues to rise, particularly among those aged 80 years and older. However, little is known about the perioperative brain health of older adults and the occurrence of short- and long-term cognitive complications associated with surgery.^50^ It is clear that postoperative cognitive recovery is a significant concern for older adults and their families, as cognitive impairment can greatly impact daily living and quality of life.^25 51 52^ The results of this study will enhance our understanding of postoperative neurocognitive function and cognitive recovery, directly benefiting many older individuals undergoing surgery and their relatives.

The evidence for non-pharmacological interventions to prevent, reduce or treat dNCR or p-NCD is limited.^53 54^ A narrative review conducted in 2024 found that a variety of interventions, including cognitive training, physical activity, transcutaneous electrical acupoint stimulation, non-invasive brain stimulation, non-pharmacological sleep improvement, music therapy, environmental modifications and multimodal combinations, can slow disease progression and enhance cognitive function after surgery.^55^ However, the review’s results primarily included the assessments of neurocognitive decline during the first week after surgery. This highlights a knowledge gap regarding non-pharmacological interventions targeting dNCR and p-NCD. The ASA Brain Health Initiative has emphasised the need for prospective studies to determine whether pre- and postoperative cognitive testing, alone or in combination with care interventions, can improve cognitive recovery, particularly among older adults.^53^ In accordance with this recommendation, we plan to assess neurocognitive performance before and after cardiac surgery using a self-administered digital test battery.

As healthcare increasingly focuses on providing specialised care for older individuals in community and primary care settings, the need to monitor symptoms remotely through digital solutions has never been more critical. Following best practices for developing and evaluating complex health interventions,^56^ we are transitioning from pilot and feasibility work to a full-scale observational trial.^57^ The results of this study will further contribute to our ongoing efforts in developing and evaluating a care intervention that includes cognitive assessment and support for patients and their close relatives.

This study is novel in its exploration of the trajectory of patient-reported cognitive symptoms after open cardiac surgery. We aim to investigate the potential relationship between cognitive symptoms and postoperative neurocognitive function, as well as how these factors affect the recovery process and daily life of older adults and their families. We expect the results to provide a scientific basis for understanding how, when and in what ways we can best support older adults in their cognitive recovery after cardiac surgery.

This study has several strengths, including its prospective design, longitudinal follow-up and collection of both objective neurocognitive measures and patient-reported outcomes. However, some limitations should be noted. We do not collect data on premorbid cognitive and social functioning, which could have provided insight into the participant’s cognitive reserve—a crucial factor to consider regarding cognitive recovery. The neurocognitive assessment is performed remotely without a test leader present, and we cannot, therefore, ensure that the participant performs the tests independently or follows the instructions. Furthermore, the Mindmore-P neurocognitive test battery lacks parallel test versions, and therefore, we cannot fully control learning effects. However, a study examining the test–retest reliability, learning effects and measures of change in Mindmore’s neurocognitive tests shows that the observed learning effects during repeated testing are minimal and within clinically acceptable limits.^58^ We have decided that patients without close relatives, or whose relatives choose not to participate, will still be included in the study. While this could be seen as a potential weakness, it is essential to acknowledge that making participation mandatory might lead us to overlook patients who are more vulnerable due to a lack of close relatives. Our primary focus remains on the patients themselves. Additionally, since we will primarily focus on including only one relative, we will provide a single perspective on the issue. In contrast, including multiple relatives as respondents does not necessarily lead to more reliable or valid data; combining responses from several relatives can create challenges in interpreting the results.^59^

The present study will address the existing knowledge gap regarding cognitive decline, contributing risk factors and the everyday experiences related to cognitive recovery following cardiac surgery. It aims to empower patients to take a more active role in their care, guide caregivers in enhancing cognitive health for older adults undergoing cardiac surgery and potentially pave the way for future interventions.
