# Supplementary material for: Exploring cognitive function and postoperative neurocognitive recovery after cardiac surgery in older adults (ECPON): a protocol for an observational study
Source: BMJ Open. 2025 Jun 22;15(6):e098208. doi: 10.1136/bmjopen-2024-098208 (PMC12184359; doi:10.1136/bmjopen-2024-098208)
Supplement: Supplementary file 1 [file bmjopen-2024-098208.reviewer_comments.pdf]

PEER REVIEW HISTORY

BMJ Open publishes all reviews undertaken for accepted manuscripts. Reviewers are asked to complete a checklist review form and are provided with free text boxes to elaborate on their assessment. These free text comments are reproduced below.

ARTICLE DETAILS

Title (Provisional)

Exploring Cognitive function and PostOperative Neurocognitive recovery after cardiac surgery in older adults (ECPON): a protocol for an observational study

Authors

Bergman, Lina; Zecevic, Ernad; Damén, Tor; Gabriela, Markovic; Martinik, Anna; Saarijärvi, Markus; Eckerblad, Jeanette; Nilsson, Ulrica

VERSION 1 - REVIEW

**Reviewer**	**1**
**Name**	**Hakiki, Bahia**
**Affiliation**	**IRCCS Fondazione Don Carlo Gnocchi**
**Date**	**13-Jan-2025**
**COI**	**None**

This authors aimed in this longitudinal observational study to investigate neurocognitive function and the cognitive symptoms experienced after cardiac surgery among >65 years adults while also capturing the perspectives of their close relatives on the recovery process.The propose to explore the trajectories of cognitive symptoms after an open cardiac surgery also interm of participation and autonomy of old patients.The protocol is well written. The introduction is well written and the gap of knowledge well presented. The aims are clearly exposed.

However, I have some major concerns exposed here:

A. I invite the authors to include some important aspects that have an impact on the cognitive state in eldery and thus should be included in the analysis.

1. In particular the pre-surgical lifestyle should be considered to estimate the cognitive reserve. This could be measured by the mean of some surrogates such as the educational level (doi: 10.1212/WNL.0000000000007036) or the occupational level (doi: 10.1080/13607863.2020.1746739.) ore bilingualism (doi: 10.3233/JAD-190397.) or by specific indexes.

2. The comorbidity impact on the cognitive state, for this some specific scales could be used (Cumulative Illness Rating Scale-Geriatric-CIRS-G) (Tekin S. Activities of daily living in Alzheimer's disease: neuropsychiatric, cognitive, and medical illness influences. Am J Geriatr Psychiatry. 2001 Winter;9(1):81-6.)

3. Some surrogates such as the presence of falls and the nutritional state couldbe used as marker of comorbidity (Fuller GF. Falls in the elderly. Am Fam Physician. 2000 Apr 1;61(7):2159-68, 2173-4. PMID: 10779256.) an should be addressed at follow-up

4. The pharmacological treatment impacting the cognitive state should be taken into account among the variables.

B. The esclusioni criteri “Participants who feel they have physical, mental, or cognitive difficulties that prevent them from completing the cognitive tests” should be better clarified since it may constitute a bias.

C. Did the authors considered who to deal with patients with social concerns who have no relatives, or will they be excluded creating a bias? This should be stated and eventually discussed in the limitation of the study. A limitation chapter should be written discussing potential weaknesses of the study including those above mentioned.

D. the authors written about "trajectories" but the type of statistical analysis the proposed do not includ IA approaches, please clarify how this will be done.

**Reviewer**	**2**
**Name**	**Butz, Marius**
**Affiliation**	**Kerckhoff Heart and Thorax Center, Bad Nauheim**
**Date**	**17-Mar-2025**
**COI**	**None**

Thank you very much for this interesting study. I think that the results will have a meaningful value for this field of research. However, I would like to ask the authors to consider the comments below.

1. Abstract; Introduction: What is the difference between neurocognitive function and cognitive symptoms? As I understand it, cognitive symptoms refer to patients' subjective perception. Would it not be more precise to speak of patients-reported/subjective cognitive symptoms or something else? This distinction should be made clearer.

2. Introduction, Line 45: The ADL criterion is also mentioned in Evered et al. (2018). Why do you cite the DSM-5 (Reference 11) here instead?

3. Risk Factors Regarding dNCR/p-NCD: Please consider including anxiety as a risk factor alongside depression.

4. Aims: In your fourth research question, you suddenly refer to "postoperative cognitive decline" (POCD). This is somewhat confusing since you previously used the terms d-NCR/p-NCD. Additionally, would it not be relevant to investigate which factors pose a risk for postoperative delirium (POD) as well?

5. Inclusion criteria: Which cardiosurgical operations are considered here? Bypass grafting, valve replacement...?

6. Cognitive Symptoms: As I understand it, you use the SwQoR-Cog for patients' subjective self-assessment of cognitive abilities. Are there specific reasons why you chose SwQoR-Cog over CFQ-s (self-assessment)? Is it superior?

7. Neurocognitive Function: Does the Mindmore test battery have parallel test versions to control for learning effects?

8. Will a trained test administrator be available only on demand via telephone or digital platform, or is it planned that they will be explicitly present via telephone or digital platform for each testing session? If the test administrator is present in the hospital, will this only apply to the preoperative assessment?

9. I also see a potential problem with the CERAD word list: How can you make sure that patients do not write down the words during the learning phase (three trials) and thereby bias the delayed recall assessment?

10. CFQ-F: You state that the CFQ-f has been translated into Swedish. Is there a reference for this, or was it translated by your working group? Should the translated CFQ-f then be validated as part of your study? And what exactly do you mean by the CFQ-f being adapted for the perioperative context?

11. Interview on Postoperative Cognitive Recovery: Why is this interview conducted only with a subsample of participants?

12. Additionally, you ask: "Have you experienced any cognitive symptoms or difficulties since the surgery?" Do you also assess whether patients have undergone other surgeries or experienced events that may have led to cognitive impairments in the meantime? If so, the question might no longer be directly related to cardiac surgery. Please discuss.

13. Qualitative Interviews: Could you describe the recruitment process for these interviews in more detail? Are there specific inclusion criteria that must be met?

14. Discussion, Line 68: You writing: "To date, there are no existing pharmacological or non-pharmacological interventions to prevent, reduce, or treat dNCR/p-NCD." This statement is misleading, as multiple studies, reviews and meta-analysis describe effective preoperative and postoperative cognitive interventions, particularly for cardiac surgery-related neurocognitive disorders. Here are some examples: doi: 10.3389/fneur.2023.1146164; doi:

10.3389/fneur.2023.1293153; doi.org/10.1007/s44254-023-00014-6. Other non-pharmacological interventions are also described in this context. See: doi: 10.3389/fneur.2024.1369821.

15. Could you please mention in your manuscript, or at least take into account for your analysis, that a different degree of preoperative anxiety and stress can be assumed in patients undergoing urgent cardiac surgery compared to elective cases. And since preoperative anxiety/stress is a risk factor for d-NCR/p-NCD, the determination of the incidence could be biased here.

VERSION 1 - AUTHOR RESPONSE

Reviewer 1	Responses and comments
Dr. Bahia Hakiki, IRCCS Fondazione Don Carlo Gnocchi<br>Comments to the Author:<br>This authors aimed in this longitudinal observational study to investigate neurocognitive function and the cognitive symptoms experienced after cardiac surgery among >65 years adults while also capturing the perspectives of their close relatives on the recovery process. The propose to explore the trajectories of cognitive symptoms after an open cardiac surgery also interm of participation and autonomy of old patients. The protocol is well written. The introduction is well written and the gap of knowledge well presented. The aims are clearly exposed. However, I have some major concerns exposed here:	Thank you for taking the time to review our manuscript. We greatly appreciate your valuable feedback and have carefully considered all your suggestions to improve our study. Below, you will find our detailed point-by-point response.
A. I invite the authors to include some important aspects that have an impact on the cognitive state in eldery and thus should be included in the analysis.<br>1. In particular the pre-surgical lifestyle should be considered to estimate the cognitive reserve. This could be measured by the mean of some surrogates such as the educational level (doi: 10.1212/WNL.0000000000007036) or the occupational level (doi: 10.1080/13607863.2020.1746739.) ore	A1. Yes, we agree that an estimate of cognitive reserve (CR) is an important aspect to consider. In our study, we do not collect all data needed to calculated CR score such as occupation, years of education, engagement in leisure activities, or a premorbid ability estimate (i.e. the individual's cognitive functioning before the onset of illness) (Megari K, Kosmidis MH. Protecting the Brain While Healing Hearts: The Protective Role of Cognitive Reserve in Cardiac Surgery. Am J Geriatr Psychiatry. 2024 Feb;32(2):195-

bilingualism (doi: 10.3233/JAD-190397.) or by specific indexes.	204.) Thus, we will not be able to assess if low CR is a predictor for dNCR/p-NCD in our analysis. This is also outside the scope of the present study, as it is neither specified in the overall aim nor in the research questions. However, we do collect data on educational level, as clarified on **page 10, line 2**. Accordingly, to a systematic review, educational level seems like the most commonly assessed cognitive reserve indicator in relation to PNCD (Feinkohl et al. Cognitive reserve and the risk of postoperative cognitive dysfunction—a systematic review and meta-analysis. Dtsch Arztebl Int 2017; 114: 110–7). Educational level will be included in the analysis when assessing risk factors for postoperative cognitive decline, and the results can be discussed in relation to literature on CR and its relationship to cognitive recovery (**page 5, line 29**).
A2. The comorbidity impact on the cognitive state, for this some specific scales could be used (Cumulative Illness Rating Scale-Geriatric-CIRS-G) (Tekin S. Activities of daily living in Alzheimer's disease: neuropsychiatric, cognitive, and medical illness influences. Am J Geriatr Psychiatry. 2001 Winter;9(1):81-6.)	A2. We will collect data on comorbidity to describe our sample. However, we do not intend to include comorbidity as an independent variable in our analysis. Although we acknowledge that this is important to investigate, this aspect is not within the scope of our aim or research questions.
A3. Some surrogates such as the presence of falls and the nutritional state could be used as marker of comorbidity (Fuller GF. Falls in the elderly. Am Fam Physician. 2000 Apr 1;61(7):2159-68, 2173-4. PMID: 10779256.) an should be addressed at follow-up	A3. Unfortunately, we do not collect data on falls or nutritional state, but we will measure frailty pre- and post-surgery. Frailty can serve as a predictor of postoperative complications and will be included in the analysis of risk factors of suffering from postoperative cognitive decline (Evered L, et al. Acute peri-operative neurocognitive disorders: a narrative review. Anaesthesia. 2022 Jan;77 Suppl 1:34-42.)
A4. The pharmacological treatment impacting the cognitive state should be taken into account among the variables.	A4. Pharmacological treatment is undoubtedly important; however, there appears to be a lack of consensus regarding the evidence for various types of anesthesia, analgesics, statins,

	corticosteroids, and their relationship to dNCR/p-NCD. The estimated sample size is 220, and we believe this sample is too small to conduct any subgroup analyses on pharmacological treatments. However, we do collect risk factors such as age, educational level, and depression, along with ASA scores and comorbidities including hypertension (Yang X et al. Identification of individuals at risk for postoperative cognitive dysfunction (POCD). Ther Adv Neurol Disord. 2022 Aug 16; 15:17562864221114356). When reporting study findings, we will provide an in-depth description of 'standard care' to describe our study setting, thereby enhancing generalizability and transferability. This will include a description of general practices in relation to pharmacological treatment for older patients undergoing open cardiac surgery.
B. The esclusioni criteri "Participants who feel they have physical, mental, or cognitive difficulties that prevent them from completing the cognitive tests" should be better clarified since it may constitute a bias.	B. Thank you for your remark. We have clarified this criterion (**page 6, line 28**). In our study, we deliberately opted for liberal inclusion criteria to ensure our observed incidence rates of cognitive decline more accurately reflect real-world figures, enhancing the study's clinical relevance. Moreover, since all patients undergo a baseline cognitive assessment, we can account for potential biases related to pre-surgical cognitive impairment.
C. Did the authors considered who to deal with patients with social concerns who have no relatives, or will they be excluded creating a bias? This should be stated and eventually discussed in the limitation of the study. A limitation chapter should be written discussing potential weaknesses of the study including those above mentioned.	C. We will not exclude patients if they do not have any relatives (or if the relatives do not want to participate). This has been clarified on page 7, lines 7-8. This could be seen as a potential weakness, but it is essential to acknowledge that making close relative participation mandatory might lead us to overlook patients who are more vulnerable due to a lack of relatives. Our primary focus remains on the patients themselves. Additionally, since we will primarily focus on including

	only one relative, we will provide a single perspective on the issue. In contrast, including multiple relatives as respondents does not necessarily lead to more reliable or valid data; combining responses from several relatives can create challenges in interpreting the results. These reflection about have been included in the discussion (**page 15, line 1-9**).
D. the authors written about "trajectories" but the type of statistical analysis the proposed do not includ IA approaches, please clarify how this will be done.	D. Longitudinal data will be analysed with mixed effect linear models to explore possible recovery trajectories. The recovery trajectory will be analysed with Statistical Process Control recommended for process analyses in healthcare related research (Benneyan et al. Statistical process control as a tool for research and healthcare improvement. Qual Saf Health Care. 2003 Dec;12(6):458-64. The statistical methods for longitudinal data is further described in methods (**page 12, line 17-23**).
**Reviewer 2**	**Response and comments**
Dr. Marius Butz, Kerckhoff Heart and Thorax Center, Bad Nauheim<br>Comments to the Author:<br>Thank you very much for this interesting study. I think that the results will have a meaningful value for this field of research. However, I would like to ask the authors to consider the comments below.	Thank you for taking the time to review our manuscript. We greatly appreciate your valuable feedback and have carefully considered all your suggestions to improve our study. Below, you will find our detailed point-by-point response.
1. Abstract; Introduction: What is the difference between neurocognitive function and cognitive symptoms? As I understand it, cognitive symptoms refer to patients' subjective perception. Would it not be more precise to speak of patients-reported/subjective cognitive symptoms or something else? This distinction should be made clearer.	1.This is a helpful suggestion for clarification. We have included "patient-reported" in both the abstract and the manuscript to enhance clarity.
2. Introduction, Line 45: The ADL criterion is also mentioned in Evered et al. (2018). Why do you cite the DSM-5 (Reference 11) here instead?	2. We have updated the information regarding the diagnostic criteria for neurocognitive disorders as follows:

	I) Subjective complaints or cognitive symptoms reported by the patient, a caregiver, or a close relative/friend;<br>II) Objective impairments or changes evaluated through neurocognitive tests;<br>III) The individual's level of functioning concerning activities of daily living (ADLs). This update refers to both the DSM-5 and Evered (**page 4, line 22-26**).
3. Risk Factors Regarding dNCR/p-NCD: Please consider including anxiety as a risk factor alongside depression.	3. In an ongoing systematic review of the association between psychological factors and postoperative cognitive decline (including delirium, dNCR, and p-NCD) in older adults, we have not found any preliminary evidence linking anxiety to these outcomes. However, we have identified a significant association between depression and delirium (PROSPERO CRD42024581115). Due to the response burden, we are therefore focusing primarily on depression. Additionally, since we are measuring health-related quality of life using the RAND 36, we have the opportunity to analyze the association between mental health and dNCR/p-NCD. This aspect has been included in the research questions (**page 5, line 30**) and methods (**page 9, line 1-3**).
4. Aims: In your fourth research question, you suddenly refer to "postoperative cognitive decline" (POCD). This is somewhat confusing since you previously used the terms d-NCR/p-NCD. Additionally, would it not be relevant to investigate which factors pose a risk for postoperative delirium (POD) as well?	4. The aim has been revised for clarity (**page 5, line 18-21**). The risk of POD is a sub-study for which we are applying for an ethical permit, but it will not be mentioned in this protocol.
5. Inclusion criteria: Which cardiosurgical operations are considered here? Bypass grafting, valve replacement…?	5. This information has been added to the manuscript (**page 6, line 18**)
6. Cognitive Symptoms: As I understand it, you use the SwQoR-Cog for patients' subjective self-assessment of cognitive abilities. Are there specific reasons why you chose SwQoR-Cog over CFQ-s (self-assessment)? Is it superior?	6. The SwQoR-Cog focuses on symptoms related to cognitive decline, such as fatigue, which is not assessed in the CFQ-s. We therefore believe that it will be superior for the purpose of measuring cognitive symptoms. However, the CFQ-for-others

	will be used in the study to assess cognitive difficulties by relatives.
7. Neurocognitive Function: Does the Mindmore test battery have parallel test versions to control for learning effects?	7. The test battery, Mindmore-P, does not have a parallel test version. However, a recently conducted test-retest reliability study (Bergman et al. Test-retest reliability, practice effects and estimates of change: A study on the Mindmore digital cognitive assessment tool. Scand J Psychol. 2025 Feb;66(1):1-14) shows that the observed learning effects in repeated testing are small and within clinically acceptable limits. Information about this limitation has been inserted in the discussion (**page 14, line 30-33**)
8. Will a trained test administrator be available only on demand via telephone or digital platform, or is it planned that they will be explicitly present via telephone or digital platform for each testing session? If the test administrator is present in the hospital, will this only apply to the preoperative assessment?	8. The test administrator will be available via telephone, digitally, or in person, depending on the specific case, particularly when assessing neurocognitive function. How the test will be administrated will be documented. This information has been inserted in the manuscript (**page 10, line 22-23**)
9. I also see a potential problem with the CERAD word list: How can you make sure that patients do not write down the words during the learning phase (three trials) and thereby bias the delayed recall assessment?	9. We cannot guarantee that patients will answer the questions or complete the tests independently. We must rely on the participants and emphasize the importance of following the guidelines for conducting the assessments. This limitation has now been added in the discussion (**page 14, line 27-29**)
10. CFQ-F: You state that the CFQ-f has been translated into Swedish. Is there a reference for this, or was it translated by your working group? Should the translated CFQ-f then be validated as part of your study? And what exactly do you mean by the CFQ-f being adapted for the perioperative context?	10. The translation information has been updated to include details about the research group that conducted it, and information about adaptation has been revised (**page 9, line 20-24**). Yes, CFQ-f will undergo further psychometric evaluation as part of the present study, as stated in the data analysis section.
11. Interview on Postoperative Cognitive Recovery: Why is this interview conducted only with a subsample of participants?	11. It is not feasible to conduct a qualitative study with 220 participants. In qualitative research, the number of participants is based on the expected amount of information required to answer the research question. Based on the concept of information power, we

	have estimated that we need to include at least 20 patients and 20 close relatives to achieve strength and richness in data collected (Malterud et al, 2016. Sample Size in Qualitative Interview Studies: Guided by Information Power. Qualitative Health Research, 26(13), 1753-1760). We have clarified this at **page 11, line 21-2.**
12. Additionally, you ask: "Have you experienced any cognitive symptoms or difficulties since the surgery?" Do you also assess whether patients have undergone other surgeries or experienced events that may have led to cognitive impairments in the meantime? If so, the question might no longer be directly related to cardiac<br>surgery. Please discuss.	12. If the patient has undergone another major surgery in the time between open-heart surgery and the interview, they will not be included in the subsample. Major surgery is defined as a surgical procedure that lasts longer than one hour in duration. This information has been inserted in the methods (**page 11, line 29-31**).
13. Qualitative Interviews: Could you describe the recruitment process for these interviews in more detail? Are there specific inclusion criteria that must be met?	13. We aim to include patients who have experienced cognitive symptoms. To identify these individuals, we have established cutoff scores for persistent cognitive symptom burden. Cognitive symptom burden is assessed using the SwQoR-Cog, with 4 non-cognitive items removed. The remaining 10 items form the cognitive subscore, ranging from 0 to 100. The threshold for symptom burden is defined as a subscore of ≥40. For inclusion in qualitative interviews, participants must exceed the cutoff (i.e. ≥40) measured at 6 and 12 weeks postoperatively. This has been added in the manuscript (**p 11, line 23-29**)
14. Discussion, Line 68: You writing: "To date, there are no existing pharmacological or non-pharmacological interventions to prevent, reduce, or treat dNCR/p-NCD." This statement is misleading, as multiple studies, reviews and meta-analysis describe effective preoperative and postoperative cognitive interventions, particularly for cardiac surgery-related neurocognitive disorders. Here are some examples: doi: 10.3389/fneur.2023.1146164; doi:	14. Pharmacological interventions have been deleted. Instead, we focus on the lack of evidence for non-pharmacological intervention. The suggested reference: doi: 10.3389/fneur.2024.1369821. is a narrative review conducted in 2024. They found that various interventions—such as cognitive training, physical activity, transcutaneous electrical acupoint stimulation, noninvasive brain stimulation, non-pharmacological sleep

10.3389/fneur.2023.1293153; doi.org/10.1007/s44254-023-00014-6. Other non-pharmacological interventions are also described in this context. See: doi: 10.3389/fneur.2024.1369821.	improvement, music therapy, environmental modifications, and multimodal combinations—can slow disease progression and enhance postoperative cognitive function. However, the review's results only included assessments of neurocognitive decline during the first week after surgery. Additionally, the only study that examined cognitive function over a one-month period targeted individuals with slow disease progression due to minor cognitive decline or Alzheimer's disease. This highlights a knowledge gap regarding non-pharmacological interventions designed to prevent, reduce, or treat delayed dNCR and p-NCD. This information has been inserted (**page 13, line 26-32, page 15, line 1-4**).
15. Could you please mention in your manuscript, or at least take into account for your analysis, that a different degree of preoperative anxiety and stress can be assumed in patients undergoing urgent cardiac surgery compared to elective cases. And since preoperative anxiety/stress is a risk factor for d-NCR/p-NCD, the determination of the incidence could be biased here.	15.Documentation about the type of surgery, planned or urgent, will be collected. Before the neurocognitive tests (Mindmore-P), the patient will assess their levels of hearing, stress, vision, and rest using a 5-point scale. This information has been inserted (**page 8, line 1-2**)
16. Definition of d-NCR/p-NCD: According to the criteria of Evered et al. (2018), the definition of d-NCR/p-NCD should also include subjective reported cognitive impairment and a restriction in the instrumental activities of daily living in addition to the objective criteria. Could you please indicate here whether you take into account the nomenclature of Evered et al. (2018) and which criteria are then exactly used for the mild and severe cases?	16. We will not set a diagnosis; rather, we aim for an objective criterion for when suffering from d-NCR/p-NCD, meaning that a decline of 1 to 2 standard deviations usually against a normative group indicates mild and >2 standard deviations for a major decline. Evered has been cited as a reference for this information in the analysis (**page 12, line 9**).

VERSION 2 - REVIEW

Reviewer 2

Name **Butz, Marius**

Affiliation **Kerckhoff Heart and Thorax Center, Bad Nauheim**

Date **17-Apr-2025**

COI

Dear authors,

thank you for taking my comments into consideration. I would like to make two minor additional suggestions:

1. You mentioned that you did not find any studies on preoperative anxiety as a risk factor for postoperative neurocognitive disorders in a systematic literature search. Maybe the following study could be helpful in this context: “Andrew MJ, Baker RA, Kneebone AC, Knight JL. Mood state as a predictor of neuropsychological deficits following cardiac surgery. J Psychosom Res. 2000;48(6):537-46.”

2. In reply to my comment 14, you state that the review you selected only describes one study that investigates the effect of a non-pharmacologic intervention on dNCR or p-NCD beyond the first postoperative week, which indicates a knowledge gap in this research area. Please take another look at this review and the other reviews I have suggested. There are indeed studies of non-pharmacologic interventions related to surgical procedures that report beneficial effects well beyond one week postoperatively. Although such studies are relatively rare, one can certainly speak of a research gap here. However, you should revise your formulation, as your current statement is not entirely accurate.

VERSION 2 - AUTHOR RESPONSE

**Reviewer: 2**<br>**Dr. Marius Butz, Kerckhoff Heart and Thorax Center, Bad Nauheim**	**Responses and comments**
Dear authors,<br><br>thank you for taking my comments into consideration. I would like to make two minor additional suggestions:<br><br>1. You mentioned that you did not find any studies on preoperative anxiety as a risk factor for postoperative neurocognitive disorders in a systematic literature search. Maybe the following study could be helpful in this context: “Andrew MJ, Baker RA, Kneebone AC, Knight JL. Mood	Thank you. We have read the study with great interest. In our ongoing review, we excluded studies published before 2014. This study dates back to 2000, which might be the reason why it didn’t catch our attention. However, we will adhere to the previously suggested measurements of potential risk factors, as 1) data collection is ongoing and 2) we carefully selected the most appropriate measures (based on previously published literature) to minimize response burden.

state as a predictor of neuropsychological deficits following cardiac surgery. J Psychosom Res. 2000;48(6):537-46.”	
2. In reply to my comment 14, you state that the review you selected only describes one study that investigates the effect of a non-pharmacologic intervention on dNCR or p-NCD beyond the first postoperative week, which indicates a knowledge gap in this research area. Please take another look at this review and the other reviews I have suggested. There are indeed studies of non-pharmacologic interventions related to surgical procedures that report beneficial effects well beyond one week postoperatively. Although such studies are relatively rare, one can certainly speak of a research gap here. However, you should revise your formulation, as your current statement is not entirely accurate.	We agree and have revised this formulation in the discussion section **(page 13, line 23 and page 14, line 1-4)**
